# *Burkholderiacenocepacia* H111 Produces a Water-Insoluble Exopolysaccharide in Biofilm: Structural Determination and Molecular Modelling

**DOI:** 10.3390/ijms21051702

**Published:** 2020-03-02

**Authors:** Barbara Bellich, Ining A. Jou, Marco Caterino, Roberto Rizzo, Neil Ravenscroft, Mustafa Fazli, Tim Tolker-Nielsen, John W. Brady, Paola Cescutti

**Affiliations:** 1 Department of Life Sciences, University of Trieste, via L. Giorgieri 1, Bdg C11, 34127 Trieste, Italy; bbellich@units.it (B.B.); rizzor@units.it (R.R.); 2 Department of Food Science, Cornell University, Ithaca, NY 14853, USA; ij56@cornell.edu (I.A.J.); marco.caterino@ukbonn.de (M.C.); jwb7@cornell.edu (J.W.B.); 3 Department of Chemistry, University of Cape Town, Rondebosch 7701, South Africa; neil.ravenscroft@uct.ac.za; 4 Costerton Biofilm Center, Department of Immunology and Microbiology, University of Copenhagen, DK-2200 Copenhagen, Denmark; magnus.m.fazli@gmail.com (M.F.); ttn@sund.ku.dk (T.T.-N.)

**Keywords:** *Burkholderia cenocepacia* H111, biofilm exopolysaccharides, NMR, molecular modelling, polysaccharide structure

## Abstract

Biofilms are a multicellular way of life, where bacterial cells are close together and embedded in a hydrated macromolecular matrix which offers a number of advantages to the cells. Extracellular polysaccharides play an important role in matrix setup and maintenance. A water-insoluble polysaccharide was isolated and purified from the biofilm produced by *Burkholderia* *cenocepacia* strain H111, a cystic fibrosis pathogen. Its composition and glycosidic linkages were determined using Gas–Liquid Chromatography–Mass Spectrometry (GLC–MS) on appropriate carbohydrate derivatives while its complete structure was unraveled by 1D and 2D NMR spectroscopy in deuterated sodium hydroxide (NaOD) aqueous solutions. All the collected data demonstrated the following repeating unit for the water-insoluble *B*. *cenocepacia* biofilm polysaccharide: [3)-α-d-Gal*p*-(1→3)-α-d-Glc*p*-(1→3)-α-d-Gal*p*-(1→3)-α-d-Man*p*-(1→]_n_ Molecular modelling was used, coupled with NMR Nuclear Overhauser Effect (NOE) data, to obtain information about local structural motifs which could give hints about the polysaccharide insolubility. Both modelling and NMR data pointed at restricted dynamics of local conformations which were ascribed to the presence of inter-residue hydrogen bonds and to steric restrictions. In addition, the good correlation between NOE data and calculated interatomic distances by molecular dynamics simulations validated potential energy functions used for calculations.

## 1. Introduction

*Burkholderia cenocepacia* belongs to the *Burkholderia Cepacia* Complex (BCC), a group of 22 closely related species that are commonly found in the environment, and in many cases have been isolated from cystic fibrosis (CF) patients. *B. cenocepacia* accounts for the majority of the clinical isolates, comprising the most virulent and transmissible strains, which are often associated with a poor clinical outcome and the development of the cepacia syndrome [1], a necrotizing and fatal pneumonia. Several virulence determinants are known for *B. cenocepacia*, e.g., iron-chelating siderophores, extracellular enzymes, surface polysaccharides and proteins, cell-to-cell signalling, and the capacity to form biofilms [2,3]. Biofilms are multicellular communities consisting of bacteria embedded in a self-produced extracellular polymeric matrix [4], mainly composed of exopolysaccharides (Epol), proteins and extracellular DNA. The biofilm matrix forms a scaffold that holds the biofilm cells together and confers enhanced tolerance to some antibiotics, desiccation, oxidizing agents, and host defenses, as recently reviewed [5,6].

Exopolysaccharides are a major component of the biofilm matrix, being particularly involved in the mechanical stability of biofilms. The macromolecular composition of the matrices depends on the bacterial species and the environmental conditions. Therefore, it is almost impossible to generalize which types of exopolysaccharides are synthesized by individual bacterial species in differing environmental conditions. Regarding *B. cenocepacia* H111, a BerA/c-di-GMP-regulated exopolysaccharide gene cluster, comprised of 12 adjacent genes essential for biofilm formation, has been identified [7,8]. Its products were hypothesized to be involved in the biosynthesis of a major exopolysaccharide that provides structural stability to the biofilms formed by *B. cenocepacia*. The exopolysaccharide encoded by the 12 gene cluster was named Burkholderia cenocepacia exopolysaccharide (Bep), and the respective genes were designated *bepA-L* [8]. With the aim of elucidating the structure of the exopolysaccharides in *B. cenocepacia* H111 biofilms, a strain of *B. cenocepacia* H111 overproducing BerA and lacking cellulose production was used to form biofilms, and a water-insoluble exopolysaccharide, named Epol H111-INS, was extracted directly from the biofilms. The insolubility of the polysaccharide is an interesting property, since biofilm matrices are not soluble in aqueous environments but rather possess a gel-like consistency. The Epol H111-INS composition and glycosidic linkages were determined using GasLiquid Chromatography–Mass Spectrometry (GLC–MS) on appropriate carbohydrate derivatives while its complete structure was elucidated by use of 1 D and 2D NMR spectroscopy recorded in deuterated sodium hydroxide (NaOD) aqueous solutions, since the base produces a limited amount of ionized hydroxyl groups which prevent polymer aggregation and promotes solubilization in water. The individual disaccharide linkages occurring in the polysaccharide primary structure have also been modelled using molecular mechanics (MM) calculations and the Ramachandran energy surface for each disaccharide has been calculated as a function of the rotation parameters around the glycosidic linkages. The results of the modelling analysis were compared with the NMR NOE data as a test of the accuracy of the MM energy functions.

## 2. Results

### 2.1. Purification and Composition Analysis of the Water-Insoluble Exopolysaccharide

The Epol H111-INS was isolated from biofilm produced by the *B. cenocepacia* Δ*bcsB*/pBerA strain [7], which was derived from *B. cenocepacia* H111, a cystic fibrosis clinical isolate [9]. BerA is a transcriptional regulator that regulates production of the Bep polysaccharide by activating transcription of the *bepA-L* genes in *B. cenocepacia* [7]. In the *B. cenocepacia* Δ*bcsB*/pBerA strain, the *berA* gene is present on the multicopy plasmid pBerA, and the BerA protein is overproduced, which results in overproduction of the Bep exopolysaccharide. The *bcsB* gene is necessary for the production of cellulose in *B. cenocepacia*. The *B. cenocepacia* Δ*bcsB*/pBerA strain is lacking the *bcsB* gene, and therefore this strain does not produce cellulose, which makes it easier to isolate the Bep polysaccharide. Biofilm was grown on nutrient-yeast extract-glycerol (NYG) agar plates and appeared as a compact wrinkled film which was peeled from each plate in one piece (Figure S1). The Epol was extracted from the matrix using 0.3 M NaOH, followed by centrifugation to remove insoluble materials, and subjected to dialysis against water in a dialysis bag, where, after reaching the equilibrium, it precipitated as a white powder. The yield was 39 mg from 4 plates. Composition data was obtained by GLC analysis of alditol acetates derivatives and it showed Gal:Glc:Man in the molar ratio 2.0:1.0:1.0. The linkage positions for the constituent sugars were determined by GLC and GLC–MS analysis of the partially methylated alditol acetate (PMAA) derivatives. GLC analysis on a HP-1 column showed that three peaks are attributed to 3-Gal, 3-Glc, and 3-Man, all in the pyranose ring conformation (Figure S2). Integration of the respective peak areas gave the following relative molar ratios 3-Gal = 2.0, 3-Glc = 1.0, 3-Man = 1.0. The absolute configuration was established to be D for all residues.

### 2.2. NMR Assignments for Epol H111-INS Repeating Unit

**Figure 1:**
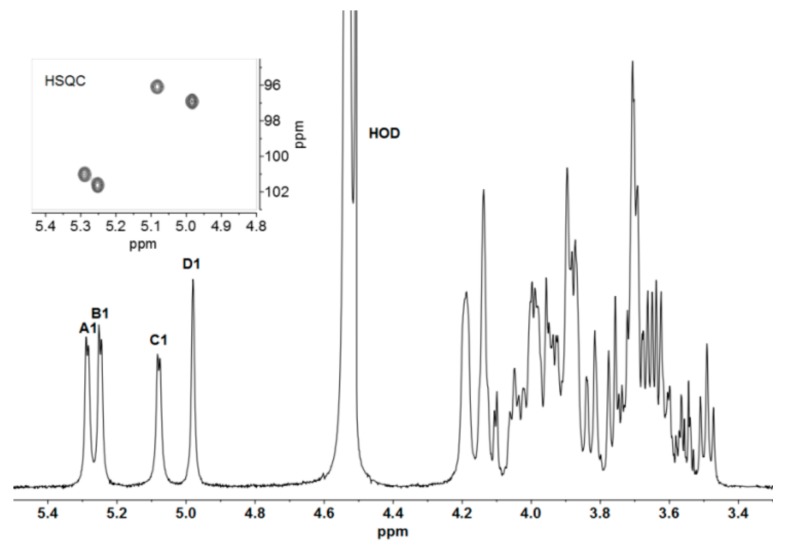
^1^H NMR spectrum recorded at 50 °C of Epol H111-INS in 0.3 M deuterated sodium hydroxide (NaOD) extracted from the biofilm of *Burkholderia cenocepacia* H111. Anomeric protons have been labelled according to the corresponding residue (**A** to **D**), as in Table 1. In the inset, the expansion of the Heteronuclear Single Quantum Coherence Adiabatic (HSQCAD) anomeric region is reported.

The repeating unit (RU) structure of Epol H111-INS was investigated at 500 MHz. The ^1^H NMR spectrum contains four anomeric signals designated **A** to **D** (Figure 1), at 5.29, 5.25, 5.08 and 4.98 ppm, and their peak area integration gave values very close to 1.0. The ^1^J_H1-H2_ values of the first three signals were in agreement with α-anomeric residues, as indicated also by their chemical shifts, while ^1^J_H1-H2_ of the resonance at 4.98 ppm was too small to be measured and attributed to H1 of Man. ^1^J_C1-H1_ were detected in a coupled Gradient Heteronuclear Single Quantum Coherence Adiabatic (gHSQCAD) experiment and the constant values measured of about 170 Hz (Table 1) are in agreement with four α-anomeric protons [10].

The tetrasaccharide RU spin systems were determined using a combination of 1D and 2D ^1^H–^1^H correlation experiments with correlations established from the four anomeric protons. The anomeric region of Correlation SpectroscopY (COSY) gave H1 to H2 for each residue (data not shown), while TOtal Correlation SpectroscopY (TOCSY) (Figure S3) gave most of the proton correlations for each of the spin systems depending on the coupling constants: H1 to H4 for α-Gal residues (**A** and **B**), H1 to H5 for α-Glc residue (**C**) and H1 to H4 for the α-Man residue (**D**). The assigned ^1^H chemical shifts for each spin system are reported in Table 1.

**Figure 2:**
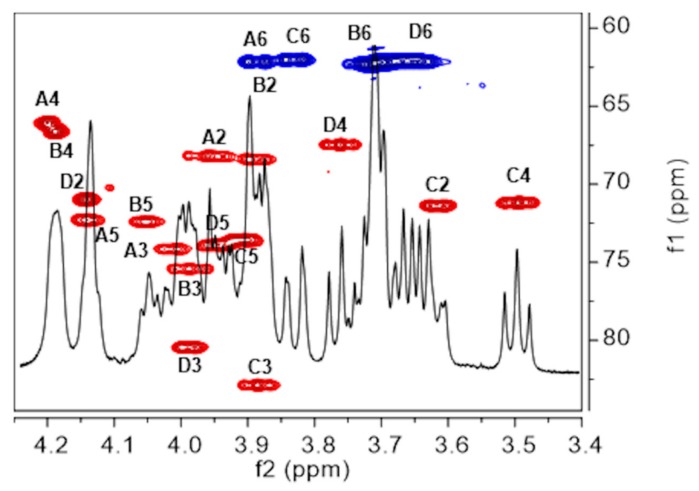
Saccharide ring region overlay of ^1^H NMR spectrum and gHSQCAD plot recorded at 50 °C of Epol H111-INS extracted from the biofilm of *B. cenocepacia*. Proton/carbon cross peaks have been labelled according to the corresponding residue (**A** to **D**), as in Table 1. Methine cross peaks are shown in red and methylene in blue.

^1^H–^13^C correlation experiments (gHSQCAD) (Figure 2) led to the assignments of the carbon atoms for each spin system. The cross peaks H5/C5 of the Gal and Man residues were determined by exclusion and in agreement with literature data [11], while H6/C6 were identified after inspection of the Gradient Heteronuclear Multiple Bond Coherence ADiabatic (gHMBCAD) plot (see next paragraph). This permitted full assignment of the ^1^H and ^13^C chemical shifts for each spin system, which are collected in Table 1. Downfield displacements of the **C**3 signals for the four spin systems compared to their shifts in the spectra of the corresponding non-substituted monosaccharides [11] demonstrated the glycosylation pattern of the RU.

**Figure 3:**
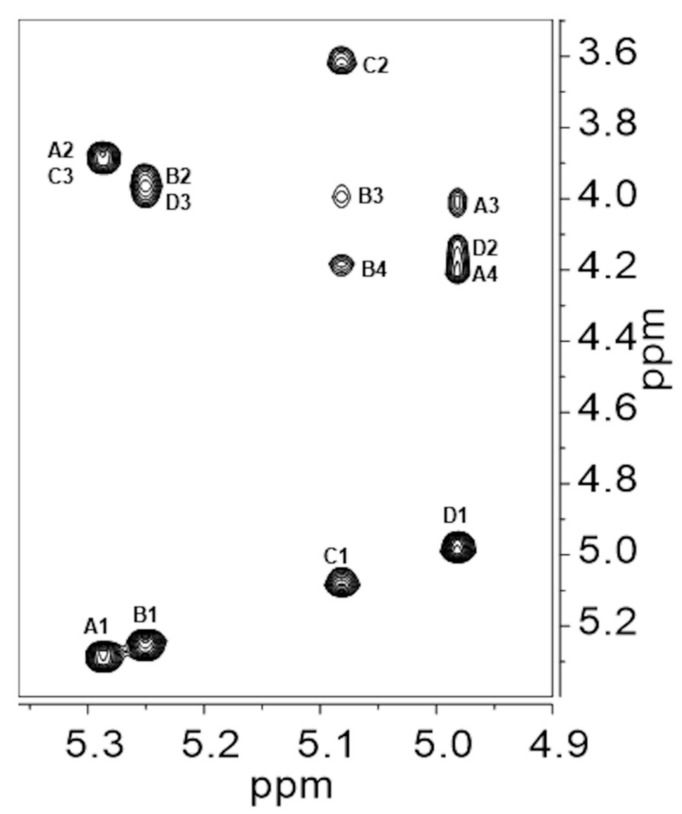
Anomeric region of the Nuclear Overhauser Effect SpectroscopY (NOESY) plot recorded at 50 °C of Epol H111-INS extracted from the biofilm of *B. cenocepacia* H111. Intra- and inter-residues connectivities are shown. Proton cross peaks have been labelled according to the corresponding residue (**A** to **D**), as in Table 1 and Table 2.

Nuclear Overhauser Effect SpectroscopY (NOESY) (Figure 3) gave intra-residue as well as inter-residue correlations and those are reported in Table 2. The following inter-residue correlations established the sequence of the monosaccharides in the RU: H1 of α-Gal (**A**) to H3 of α-Glc (**C**), H1 of α-Gal (**B**) to H3 of α-Man (**D**), H1 of α-Glc (**C**) to H3 of α-Gal (**B**) and H1 of α-Man (**D**) to H3 of α-Gal (**A**). The two latter correlations were weaker than those between H1 of α-Glc (**C**) and H4 of α-Gal (**B**), and between H1 of α-Man (**D**) and H4 of α-Gal (**A**), as evidenced in the vertical traces of the NOESY plot (Figure S4), thus suggesting a smaller distance between protons 1 and 4 with respect to the protons across the glycosidic bonds, which is usually the shortest HH distance in disaccharides. These experimental findings were confirmed by molecular modelling calculations (see next section).

**Figure 4:**
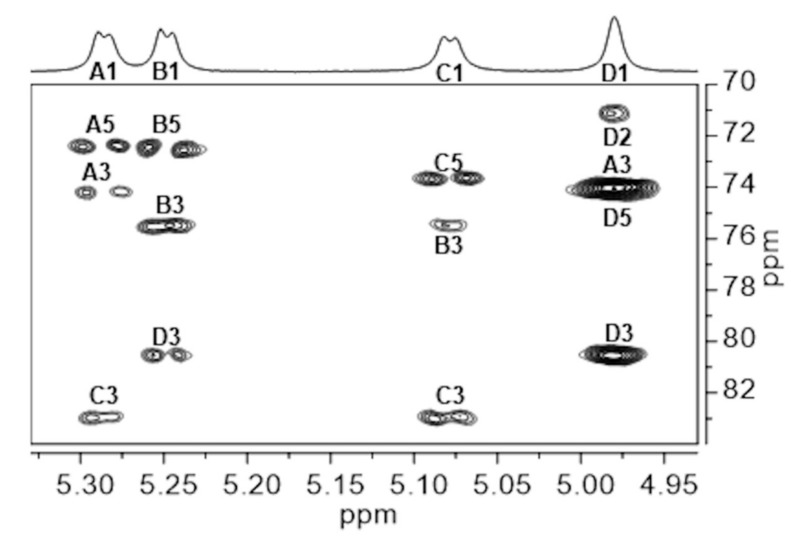
Expansion of the gHMBCAD plot recorded at 50 °C of Epol H111-INS extracted from the biofilm of *B. cenocepacia* H111. Intra- and inter-residue correlations have been labelled according to the corresponding residue (**A** to **D**), as in Table 1.

A gHMBCAD experiment gave useful intra-residue correlations which confirmed the monosaccharides chemical shift assignments and provided H6/C6 for each spin system through correlation with the respective H-4 (data not shown). Moreover, starting from each anomeric proton (Figure 4), inter-residue cross peaks confirmed the sequence information deduced from the NOESY plot: H1 of α-Gal (**A**) to C3 of α-Glc (**C**) at 82.9 ppm, H1 of α-Gal (**B**) to C3 of α-Man (**D**) at 80.4 ppm, H1 of α-Glc (**C**) to C3 of α-Gal (**B**) at 75.5 ppm and H1 of α-Man (**D**) to C3 of α-Gal (**A**) at 74.1 ppm.

In conclusion, all the experimental data collected demonstrated that the water-insoluble biofilm polysaccharide produced by *B. cenocepacia* H111 has a tetrasaccharide repeating unit with the following structure:

**A**   **C**   **B**   **D** [3)-α-D-Gal*p*-(1→3)-α-D-Glc*p*-(1→3)-α-D-Gal*p*-(1→3)-α-D-Man*p*-(1→]_n_

### 2.3. Ramachandran Conformational Maps and Molecular Dynamics Simulations

**Figure 5:**
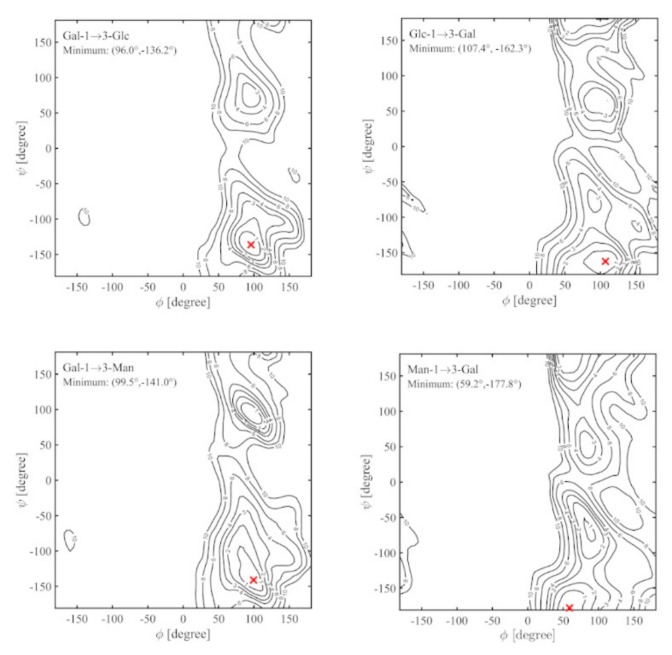
Fully relaxed conformational energy maps for each of the four linkages in the Epol H111-INS polysaccharide. The global minimum is shown with a red “x”. Contour levels are indicated in kcal/mol above the global minima at levels 1, 2, 3, 4, 6, 8, and 10.

The fully relaxed conformational energy map of each of the four disaccharide linkages found in the Epol H111-INS polysaccharide were computed and are shown in Figure 5. As these disaccharides are all of the (1→3) linkage type, there are similarities between the maps, where the majority of the allowed *ф* values are between 0° and 180°, regardless of the *ψ* values. Upon closer examination, the maps can be subdivided into two groups: those where the first (i.e., non-reducing end) sugar is Gal, and those where the second, reducing-end sugar is Gal.

**Figure 6:**
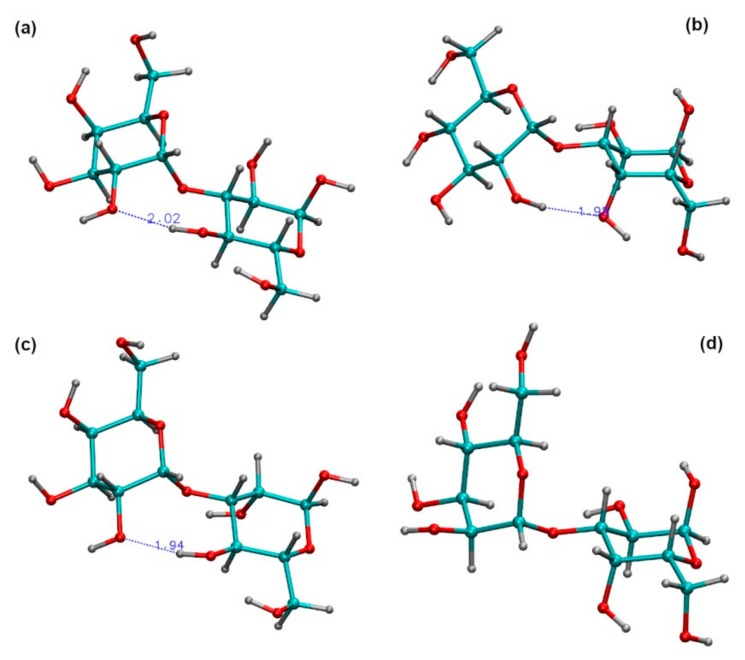
The global minimum energy structures for (**a**) the *α*-D-Gal*p*-(1→3)-*α*-D-Glc*p* disaccharide linkage, indicating the hydrogen bond between O2 of galactose and OH4 of glucose; (**b**) the *α*-D-Glc*p*-(1→3)-*α*-D-Gal*p* disaccharide linkage, indicating the hydrogen bond between OH2 of glucose and O4 of galactose; (**c**) the *α*-D-Gal*p*-(1→3)-*α*-D-Man*p* disaccharide linkage, indicating the hydrogen bond between O2 of galactose and OH4 of mannose; (**d**) the *α*-D-Man*p*-(1→3)-*α*-D-Gal*p* disaccharide linkage where no hydrogen bond can be formed.

For the first group of disaccharides, as shown on the left of Figure 5, the locations of the global minima are almost the same, with differences of less than 5° in their *ф* and *ψ* angles. This is as expected since glucose and mannose differ only in the configuration of the C2 hydroxyl group. The similarity in their global minima shows that the orientation of the C2 hydroxyl group for these linkages is not a major contributor to the torsional energy. By examining the structures of these disaccharides at their global minima it can be seen that both can form a hydrogen bond between the O2 atom of the galactose and the OH4 atom of either the glucose (Figure 6a) or mannose (Figure 6c) residue, where the distance between these two atoms is approximately 2 Å. In addition, both of these disaccharides show a second energy minimum at approximately *ф*~100° and *ψ*~80°−100°.

The other two disaccharide linkage types, where the second (reducing-end) sugar is galactose, are on the right of Figure 5. In both of these maps, there are three low energy “valleys,” with the lowest global minimum positioned in the same general valley, located between *ψ*~−150° and 150°, but at different *ф* and *ψ* values, and with the general shape of the valley being different. In the case of Glc(1→3)Gal, the structure corresponding to the global minimum energy, shown in Figure 6b, is stabilized by hydrogen bonding between the OH2 of the glucose residue and O4 of the galactose. For the Man(1→3)Gal disaccharide, this particular hydrogen bond is not possible, because the C2 hydroxyl group of mannose is on the opposite side Figure 6d. There were no other possible hydrogen bonds identified for Man(1→3)Gal. In all, three of the four disaccharide linkages of Epol H111-INS form inter-molecular hydrogen bonds and one linkage does not.

**Figure 7:**
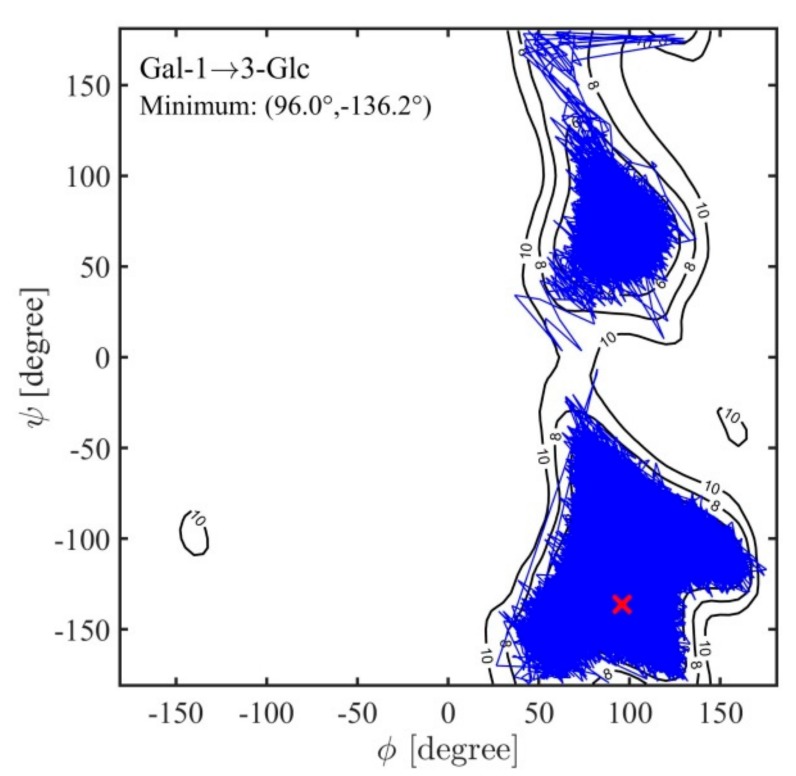
An example of a 1 μs molecular dynamics (MD) trajectory on top of the calculated fully relaxed Ramachandran conformational energy map for the disaccharide α-D-Gal*p*-(1→3)-α-D-Glc*p* whose original energy map is shown in Figure 5 top left.

**Figure 8:**
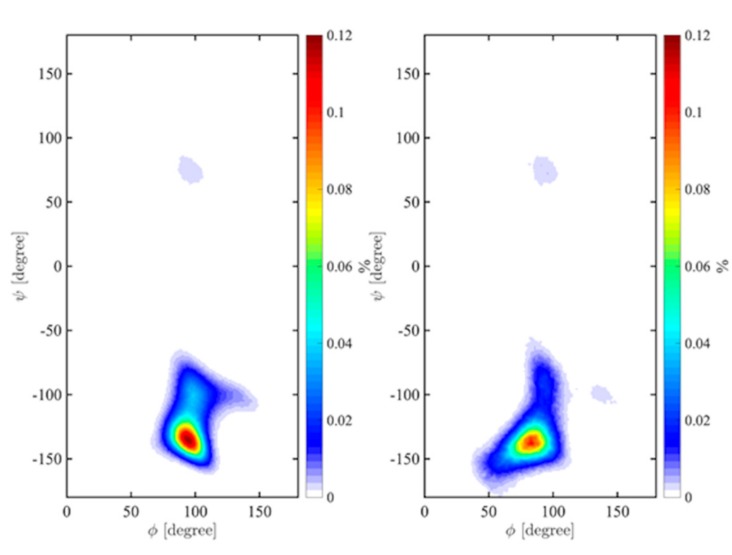
Probability density maps for α-D-Gal*p*-(1→3)-α-D-Glc*p* in vacuum (**left**) and in explicit solvent (**right**) after a 1 μs MD simulation at 300 K. Negative *ф* are neglected for readability as no point occurs in that region, in agreement with fully relaxed maps.

Molecular dynamics simulations in vacuum showed very good agreement with the calculated Ramachandran maps (as an example see Figure 7 showing the Gal(1→3)Glc case). Simulations were also performed in explicit water to study the effects of water on the conformation of these disaccharides and any shift of the minima due to solvation (Figure 8 and Figures S5–S7). Hydration slightly shifted the highest density well to lower *ф* angles for all four of the disaccharide linkages (Table 3). Upon hydration, the Gal(1→3)Glc (Figure 8) still featured a single very-high density well centered at approximately (82°, −137°) and a broader low-density distribution than in vacuum, extending to (*ф*, *ψ*) values of ~(50°, −150°). The shoulder in the density distribution approximately (140°, −100°) became a shallow isolated minimum with a low occupation probability in solution, while the valley extending to less negative *ψ* values extended a bit further, up to approximately (100°, −75°), with the latter sampled during two major transitions in explicit solvent (Figure 8).

The highest occupancy density for the Glc(1→3)Gal linkage was found to be shifted in solution along *ф* to 67° (from 99° in vacuum), while for this linkage, the hydration effect on *ψ* was small (~2°). The large, low-density well in the positive *ф*, *ψ* field at (50°, 60°) was restricted in water (Figure S5). Similarly, hydration impacted the Gal(1→3)Man linkage, as the highest-density *ф* well in water was found at ~69°, shifted from 94° in vacuum. The low-density region in the positive range of the vacuum surface at approximately (100°, 100°) was no longer accessible in water (Figure S6). Man(1→3)Gal was the only dimer showing a larger shift in *ψ* than in *ф* to (60°, −174°) from (68°, −160°) in vacuum. The low-density region centered at approximately (*ф*, *ψ*) 90°, −75° was again disfavoured in water (Figure S7).

Table 3 lists all of the interatomic distances relevant to the NMR experiments as calculated from the MD simulations of each of the linkages in the Epol H111-INS repeating unit in aqueous (TIP4P) solution at room (300 K) temperature (Figures S8–S11). Among the calculated interatomic distances, particularly relevant are H1-‘H3 and H1-‘H4 (the apostrophe indicates the reducing end residue) for the two disaccharides Man(1→3)Gal and Glc(1→3)Gal, because, contrary to what is usually found, both NOE experimental data and MD simulation indicated smaller values for H1-‘H4 than for the two protons across the glycosidic linkages. At the same time, the distances H1-‘H3 in the two disaccharides Gal(1→3)Glc and Gal(1→3)Man were found to be shorter than H1-‘H4 with both approaches, as expected for 1→3 glycosidic linkages. The consistency between NOE data and calculated interatomic distances by MD simulations validated that the simulations are in very good agreement with the NMR experimental approach.

## 3. Discussion

The data reported in the present manuscript showed the presence of a water-insoluble exopolysaccharide in the biofilm formed by *B. cenocepacia* H111. To the best of our knowledge, the structure of the Epol H111-INS is novel, not only among the BCC species, but also among bacteria in general. It is also extremely interesting that this exopolysaccharide is water-insoluble, especially considering that the few known water-insoluble polysaccharides are either *β*-glucans (cellulose and schizophyllan), and its *N*-acetylated derivative (chitin), or *β*-mannans.

It is satisfying that the experimental NMR results are completely consistent with the interatomic distances calculated from both the vacuum conformational energy maps and the conformational densities, or probabilities, calculated from the MD simulations in aqueous solution, since the shifts in conformation induced by solvent interaction are sufficiently small that they are also completely consistent with the NOE limits. This agreement can be taken as additional validation of the modelling results and will lend confidence to the results expected from simulations underway of biofilms constructed from these polymers, where comparable experimental data will be harder to obtain.

It is also worth stressing that three out of the four disaccharides composing the polymer are characterized by the presence of inter-residue hydrogen bonds, while the fourth one, Man(1→3)Gal, does not exhibit this feature. However, its local conformation seems to be rather restricted as suggested by both NOE and modelling evidences that point to a shorter H1-‘H4 interatomic distance with respect to the expected H1-‘H3 one. The presence of restricted conformational freedom, either because of the presence of hydrogen bonds or for steric factors in all the polymer backbone segments, leads to a rigid polymer chain which might explain its tendency to aggregate with subsequent water insolubility. As generally depicted for the molecular model of the biofilm matrix, macromolecular aggregation is required to setup the scaffold in which microbial cells are embedded and to constitute a network allowing small molecules (nutrients and others) to diffuse through it.

Bep production in *B. cenocepacia* is upregulated by high intracellular levels of the signaling molecule c-di-GMP via increased activity of the BerA transcriptional regulator [7,12]. The intracellular c-di-GMP level in bacteria is regulated by diguanylate cyclases and phosphodiesterases in response to various environmental cues [13]. Recent work suggests that the conditions in the lungs of the human host lead to increased cellular c-di-GMP levels in *Burkholderia* species [14], which in *B. cenocepacia*, will promote Bep production and biofilm formation. Therefore, it is likely that the Bep exopolysaccharide is of clinical relevance.

## 4. Materials and Methods

### 4.1. Bacterial Strain, Biofilm Production and Polysaccharide Purification

The Δ*bcsB*/pBerA strain [7] was derived from *B. cenocepacia* H111, a clinical isolate from a cystic fibrosis patient [9]. The strain Δ*bcsB*/pBerA contains the plasmid pBcam1349 (pBerA), composed of pBBR1MCS2 with the *berA* gene inserted in the BamHI/XbaI sites and with a deletion of the gene *bcal1389* (designated *bcsB* for Bacterial cellulose synthase subunit B)*,* the first gene in the genetic cluster devoted to cellulose biosynthesis.

Bacteria were spread from a −80 °C stock culture directly onto 4 agar plates containing the nutrient-yeast extract-glycerol (NYG) medium (0.5% peptone, 0.3% yeast extract, 2% (*w*/*v*) glycerol, and 1.5% agar) and grown for 4 days at 30 °C. The developed biofilm was peeled off the plates and placed in 50 mL falcon test tubes with 0.3 M NaOH, left shaking for 3 h at 8 °C and subjected to centrifugation at 22,400× *g* at 4 °C for 30 min. After the supernatant was extensively dialyzed against water, a white precipitate formed in the dialysis bag which was then recovered by centrifugation at 1900× *g* at 4 °C for 30 min. The precipitate was washed with a solution of ethanol:water 4:1 (v:v); the supernatant was removed after centrifugation at 1900× *g* at 4 °C for 30 min, and the precipitate was dried under N_2_. It resulted to be water-insoluble.

### 4.2. General Procedures

Native and permethylated polysaccharides were hydrolyzed with 2 M trifluoroacetic acid (TFA) at 125 °C for 1 h. Alditol acetates were prepared as previously described [15]. Permethylation of the Epol H111-INS was achieved following the protocol by Harris [16].

Analytical GLC was performed on a Perkin-Elmer Autosystem XL gas chromatograph equipped with a flame ionization detector and using He as carrier gas. An HP-1 capillary column (Agilent Technologies, 30 m) was used to separate alditol acetates (temperature program: 3 min at 150 °C, 150–270 °C at 3 °C/min, 2 min at 270 °C), PMAA (temperature program: 1 min at 125 °C, 125–240 °C at 4 °C/min, 2 min at 240 °C), and trimethylsilyl (TMS) (+)-2-butyl glycosides, for the determination of the absolute configuration of the sugar residues [17], (temperature program: 1 min at 50 °C, 50–130 °C at 45 °C/min, 1 min at 130 °C, 130–200 °C at 1 °C/min, 10 min at 200 °C). GLC–MS analyses were carried out on an Agilent Technologies 7890A gas chromatograph coupled to an Agilent Technologies 5975C VL MSD, using the same temperature programs reported above.

### 4.3. NMR Experiments

The polysaccharide was exchanged twice with 99.9% D_2_O by lyophilization, dissolved in 0.6 mL of 0.3 M NaOD in 99.96% D_2_O and introduced into a 5 mm NMR tube for data acquisition. Acetone (diluted 1:100 in D_2_O) was used as external reference in a coaxial tube and set at 2.225 ppm for ^1^H and 31.07 ppm for ^13^C. Spectra were recorded on a 500 MHz Varian spectrometer operating at 50 °C, after setting the proper pw90° pulse. 2D experiments were performed using standard pulse sequences and pulsed field gradients for coherence selection when appropriate. gHSQCAD spectra were recorded using 145 Hz (for directly attached ^1^H–^13^C correlations) and the gHMBCAD experiment optimized for a coupling constant of 8 Hz (for long-range ^1^H–^13^C correlations). TOCSY spectra were acquired using 150 ms spin-lock time and a 1.2 s relaxation time. NOESY experiments were recorded with 200 ms mixing time and a 1.2 s relaxation time. NMR spectra were processed using MestreNova software.

### 4.4. Molecular Modelling

Adiabatic vacuum Ramachandran conformational energy maps were prepared for each of the disaccharide linkages in the Epol H111-INS polymer repeat unit by exhaustive energy minimization of every possible combination of all internal degrees of freedom, other than ring shape (^4^C_1_ for all sugar residues), for every point on a 20° × 20° grid over the full 360° range of both *ф* and *ψ* for each case. These maps were calculated using the CHARMM molecular mechanics program [18,19] and the CHARMM36 force field parameters for carbohydrates [20,21], using procedures described previously [22]. Note, however, for reasons described previously, the angles *ф* and *ψ* were defined as O5-C1-O3′-C3′ and C1-O3′-C3′-C2′, using heavy atoms rather than the proton-based definitions used in NMR work, in order to avoid computational artefacts. These angle values can be approximately converted to the proton-based definitions of these angles by subtracting 120° from each.

Molecular dynamics simulations of these disaccharides, both in vacuum and in aqueous (TIP4P) solution, were also conducted at a constant pressure of 1 atm and a constant temperature of 300 K, again using the CHARMM program and CHARMM36 carbohydrate parameters, following protocols described previously.

## Figures and Tables

**Table 1:** ^1^H and ^13^C chemical shift assignments of the Epol H111-INS in 0.3 M NaOD.

Residue ^1^		Chemical Shift (ppm) ^2^					
		1	2	3	4	5	6
**A** [171]	^1^H	5.29	3.89	4.02	4.20	4.14	3.89
**→3)-α-D-Gal*p*-(1→**	^13^C	101.0	68.4	74.1	66.1	72.3	62.2
**B** [171]	^1^H	5.25	3.95	3.98	4.18	4.05	3.71
**→3)-α-D-Gal*p*-(1→**	^13^C	101.6	68.2	75.5	66.6	72.4	62.3
**C** [170]	^1^H	5.08	3.62	3.89	3.50	3.91	3.83
**→3)-α-D-Glc*p*-(1→**	^13^C	96.1	71.4	82.9	71.2	73.6	62.0
**D** [169]	^1^H	4.98	4.14	3.98	3.76	3.94	3.66
**→3)-α-D-Man*p*-(1→**	^13^C	96.9	71.1	80.4	67.5	73.9	62.1

^1^ J_C1,H1_ in square brackets. ^2^ Chemical shifts relative to external acetone (2.225 ppm for ^1^H and 31.07 ppm for ^13^C).

**Table 2:** Intra- and inter-residue Nuclear Overhauser Effect (NOE) contacts detected starting from the anomeric protons of the NOESY spectrum of Epol H111-INS in 0.3 M NaOD.

Proton/ppm.	NOE Contacts (ppm)	Assignment
**A**1/5.29	3.89	**A**2
	3.89	**C**3
**B**1/5.25	3.95	**B**2
	3.98	**D**3
**C**1/5.08	3.62	**C**2
	3.98	**B**3
	4.18	**B**4
**D**1/4.98	4.14	**D**2
	4.02	**A**3
	4.20	**A**4

**Table 3:** Highest-density (*ф*, *ψ*) points on the Ramachandran energy maps in vacuum averaged over 1 μs of trajectory simulation time and over 100 ns of simulation time in explicit TIP4P solvent at 300 K, and the NMR-relevant inter-residue distances averaged over the solution simulations, for all of the linkages found in the Epol H111-INS repeating unit.

Disaccharide	Highest Density ^1^		Average Distance (Å) ^2^				
	Vacuum (*ф*, *ψ*)	TIP4P (*ф*, *ψ*)	H1-‘H3	H1-‘H4	H1-‘H5	H2-‘O2	H1-‘C3
α-d-Gal*p*-(1→3)-α-d-Glc*p*	94 | -135	82 | -137	2.38 (0.33)	3.79 (0.40)	4.38 (0.30)	5.68 (0.71)	2.57 (0.12)
α-d-Glc*p*-(1→3)-α-d-Gal*p*	99 | -160	67 | -162	2.73 (0.34)	2.39 (0.41)	4.28 (0.25)	5.60 (0.74)	2.63 (0.12)
α-d-Gal*p*-(1→3)-α-d-Man*p*	94 | -136	69 | -130	2.34 (0.21)	3.97 (0.31)	4.40 (0.27)	5.41 (0.78)	2.58 (0.10)
α-d-Man*p*-(1→3)-α-d-Gal*p*	68 | -160	60 | -174	2.75 (0.33)	2.38 (0.39)	4.30 (0.25)	6.61 (0.32)	2.64 (0.12)

^1^ Over a 1 μs simulation in vacuum and 100 ns in TIP4P. ^2^ Averaged over the 100 ns simulations in explicit solvent. Standard deviation in round brackets.

## Supplementary Materials

Supplementary materials can be found at https://www.mdpi.com/1422-0067/21/5/1702/s1.

## Abbreviations

1D NMR	One-Dimensional Nuclear Magnetic Resonance
2D NMR	Two-Dimensional Nuclear Magnetic Resonance
BCC	*Burkholderia Cepacia* Complex
Bep	Burkholderia cenocepacia exopolysaccharide
CF	Cystic fibrosis
COSY	Correlation SpectroscopY
Epol	Exopolysaccharide
Gal	Galactose
gHMBCAD	Gradient Heteronuclear Multiple Bond Coherence ADiabatic
gHSQCAD	gradient Heteronuclear Single Quantum Coherence ADiabatic
Glc	Glucose
GLC	Gas–Liquid Chromatography
GLC–MS	Gas–Liquid Chromatography–Mass Spectrometry
Man	Mannose
MD	Molecular dynamics
MM	Molecular mechanics
NaOD	Deuterated sodium hydroxide
NaOH	Sodium hydroxide
NOE	Nuclear Overhauser Effect
NOESY	Nuclear Overhauser Effect SpectroscopY
NYG	Nutrient-yeast extract-glycerol
PMAA	Partially methylated alditol acetates
RU	repeating unit
TFA	Trifluoroacetic acid
TMS	Trimethylsilyl
TOCSY	TOtal Correlation SpectroscopY
