# Supplementary material for: Burkholderiacenocepacia H111 Produces a Water-Insoluble Exopolysaccharide in Biofilm: Structural Determination and Molecular Modelling
Source: Int J Mol Sci. 2020 Mar 2;21(5):1702. doi: 10.3390/ijms21051702 (PMC7084887; doi:10.3390/ijms21051702)
Supplement: Supplementary file 1 [file ijms-21-01702-s001.pdf]

Burkholderia cenocepacia H111 Produces a Water-Insoluble Exopolysaccharide in Biofilm: Structural Determination and Molecular Modelling.

Barbara Bellich[1], Ining A. Jou[2], Marco Caterino[2], Roberto Rizzo[1], Neil Ravenscroft[3], Mustafa Fazli[4], Tim Tolker-Nielsen[4], John W. Brady[2*], Paola Cescutti[1*]

[1] Department of Life Sciences, University of Trieste, via L. Giorgieri 1, Bdg C11, 34127 Trieste, Italy

[2] Department of Food Science, Cornell University, Ithaca, NY 14853, USA

[3] Department of Chemistry, University of Cape Town, Rondebosch 7701, South Africa

[4] Costerton Biofilm Center, Department of Immunology and Microbiology, University of Copenhagen, DK-2200 Copenhagen, Denmark.

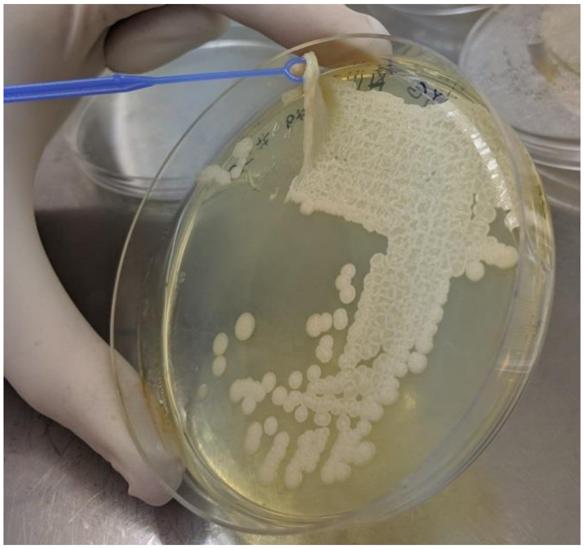

Figure S1. *Burkholderia cenocepacia* H111 biofilm developed on nutrient-yeast extract-glycerol agar plates.

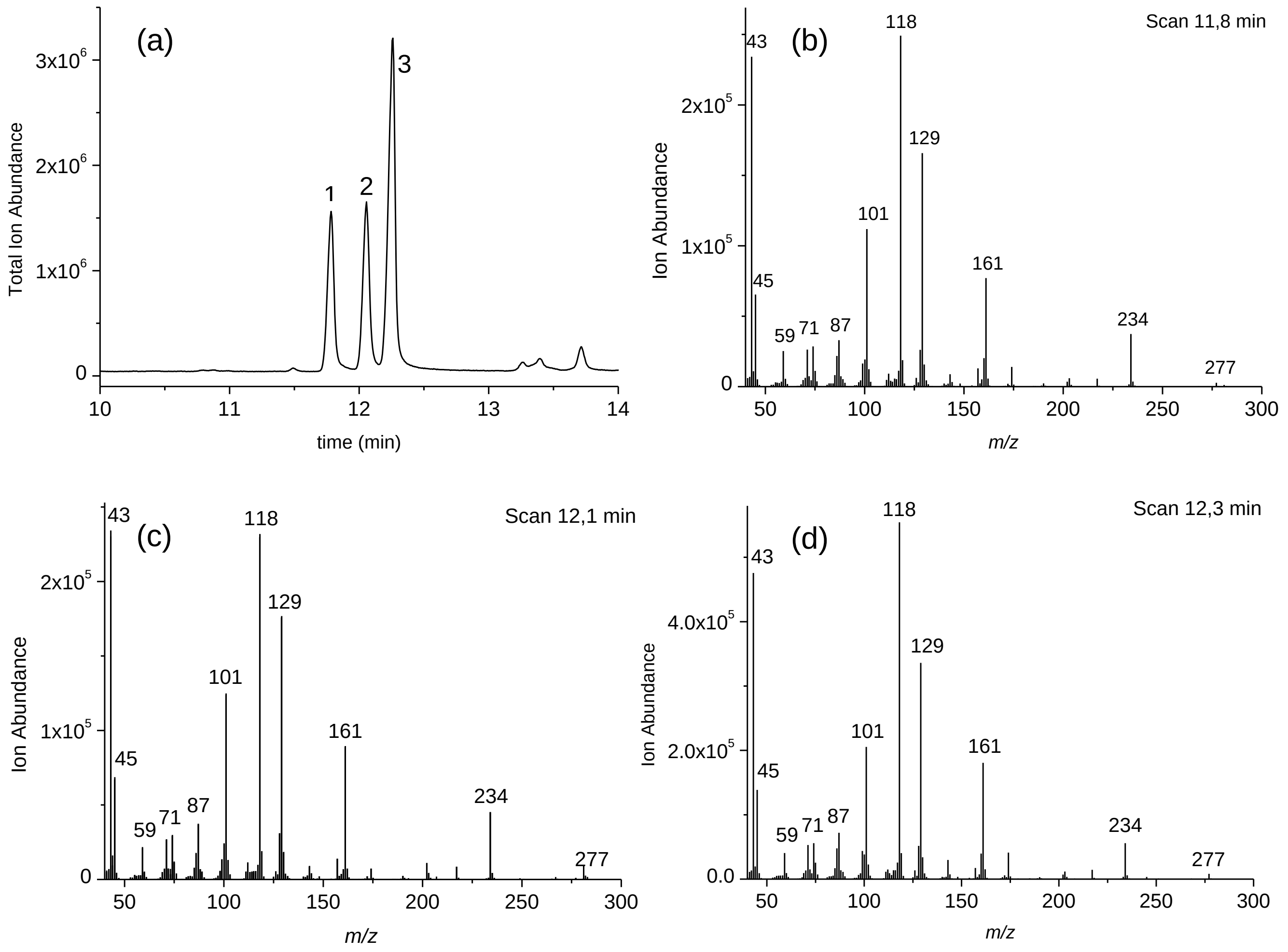

Figure S2. GLC-MS analysis of the partially methylated alditol acetate mixture obtained after derivatisation of the Epol H111-INS: (a) Total Ion Chromatogram; (b) e.i. mass spectrum of peak 1 shown in (a) and attributed to 3-linked Glc; (c) e.i. mass spectrum of peak 2 shown in (a) and attributed to 3-linked Man; (d) e.i. mass spectrum of peak 3 shown in (a) and attributed to 3-linked Gal.

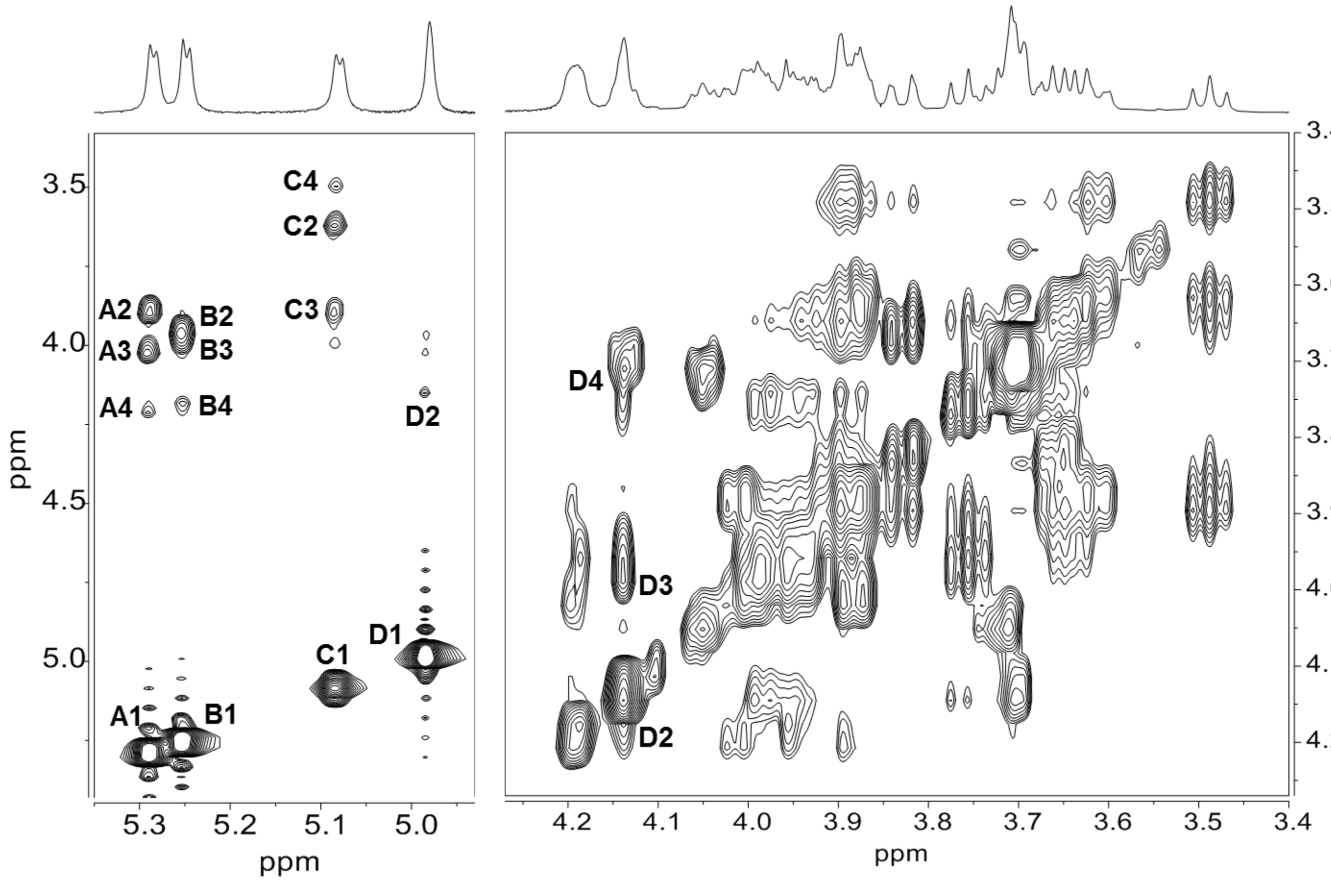

Figure S3. Expansion of the TOCSY anomeric region of Epol H111-INS recorded at 500 MHz and 50 °C. Crosspeaks have been labelled according to the corresponding residue (**A** to **D**, see Table 1).

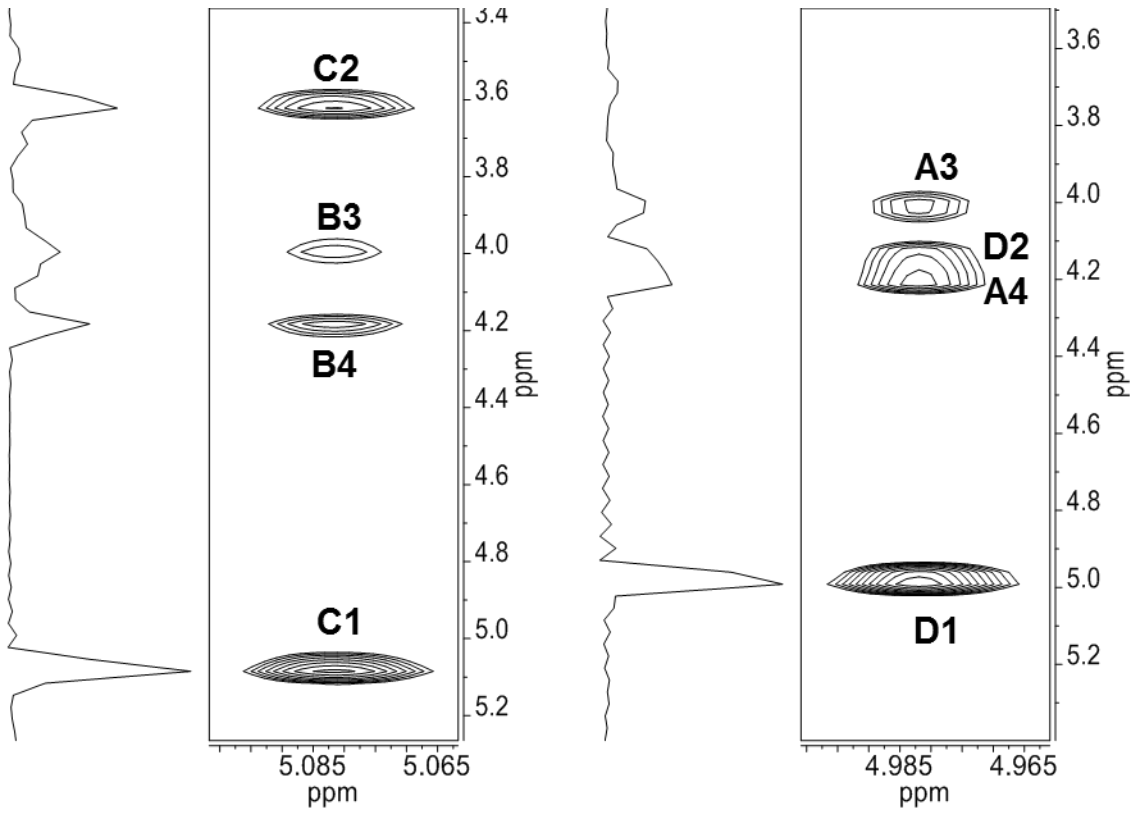

Figure S4. Anomeric region of the NOESY plot of the Epol H111-INS showing NOE connectivities and vertical traces for α-Glc (**C**1) (left) and α-Man (**D**1) (right). Crosspeaks have been labelled according to the corresponding residue (**A** to **D**, see Table 1).

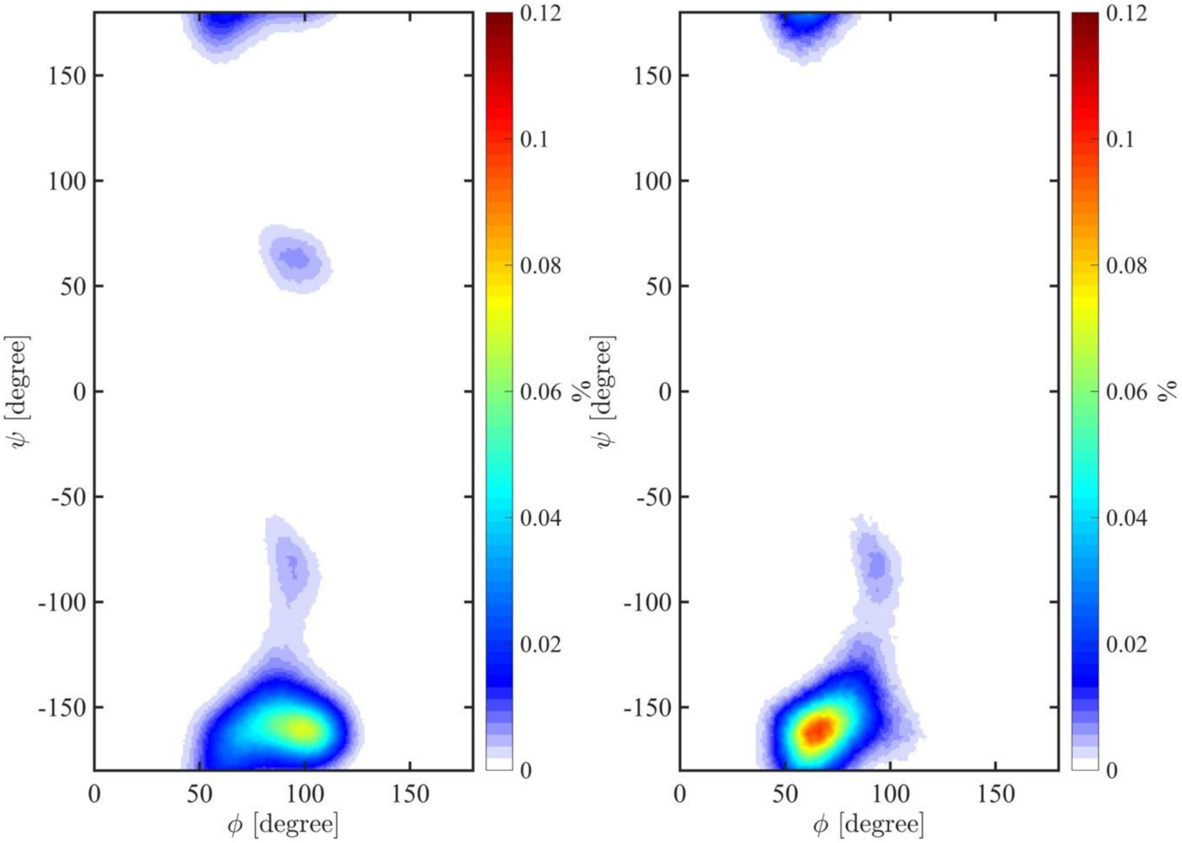

Figure S5. Probability density maps for α-D-Glc*p*-(1→3) α-D-Gal*p* in vacuum (left 1 μs) and in explicit solvent (right- 100 ns) at 300 K. Negative ϕ are neglected for readability as no point occurs in that region, in agreement with fully relaxed maps.

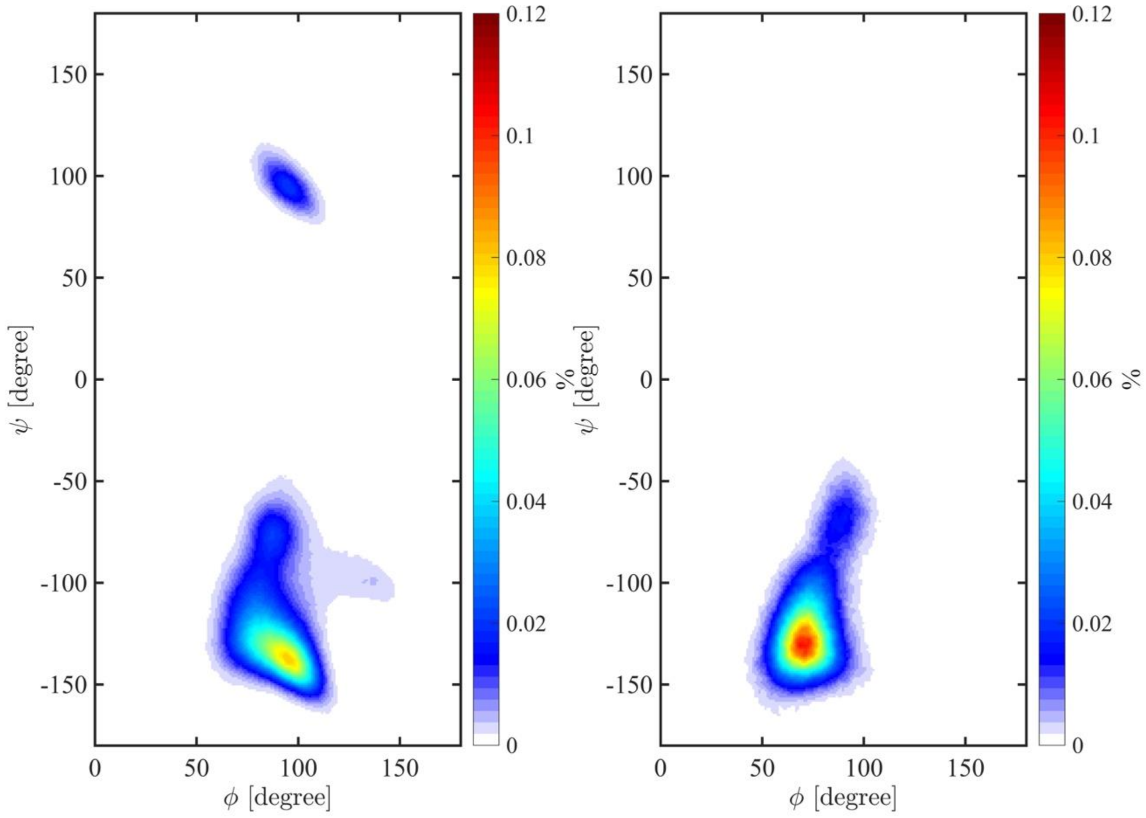

Figure S6. Probability density maps for α-D-Gal*p* (1→3)-α-D-Man*p* in vacuum (left 1 μs) and in explicit solvent (right- 100 ns) at 300 K. Negative ϕ are neglected for readability as no point occurs in that region, in agreement with fully relaxed maps

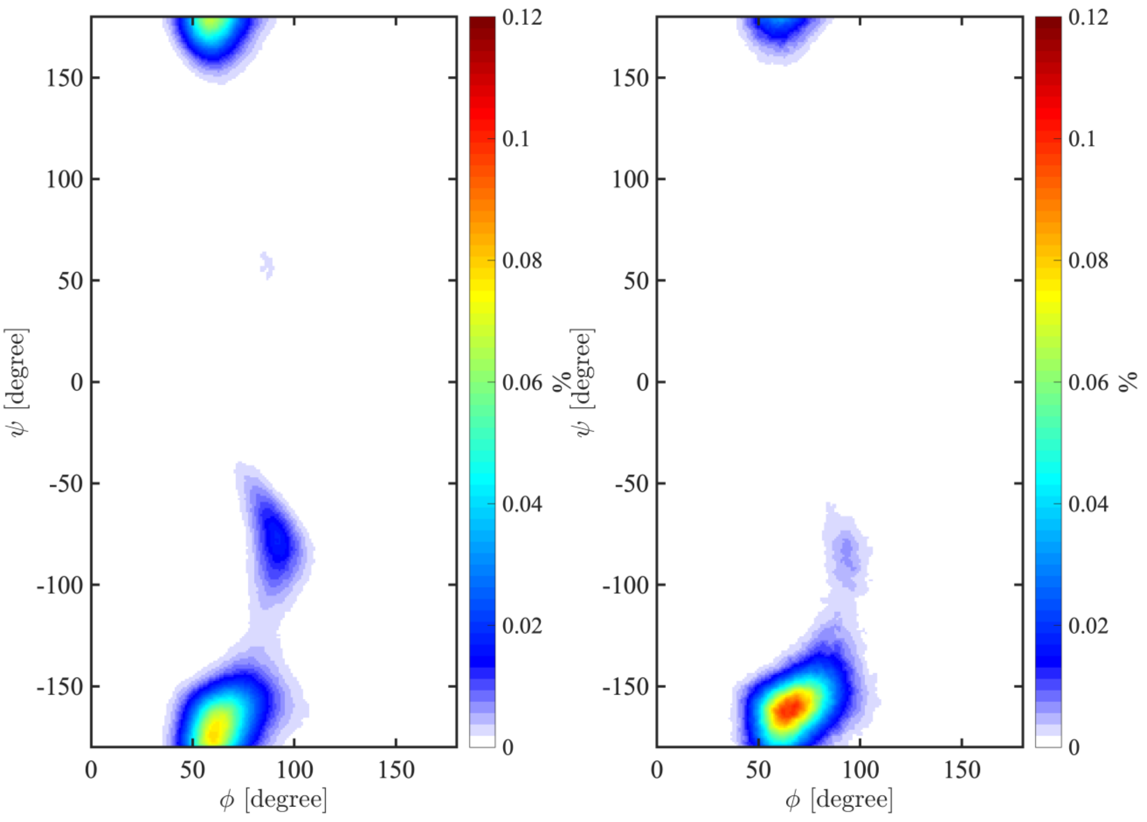

Figure S7. Probability density maps for α-D-Man*p*-(1→3)-α-D-Gal*p* in vacuum (left 1 μs) and in explicit solvent (right- 100 ns) at 300 K. Negative *ϕ* are neglected for readability as no point occurs in that region, in agreement with fully relaxed maps.

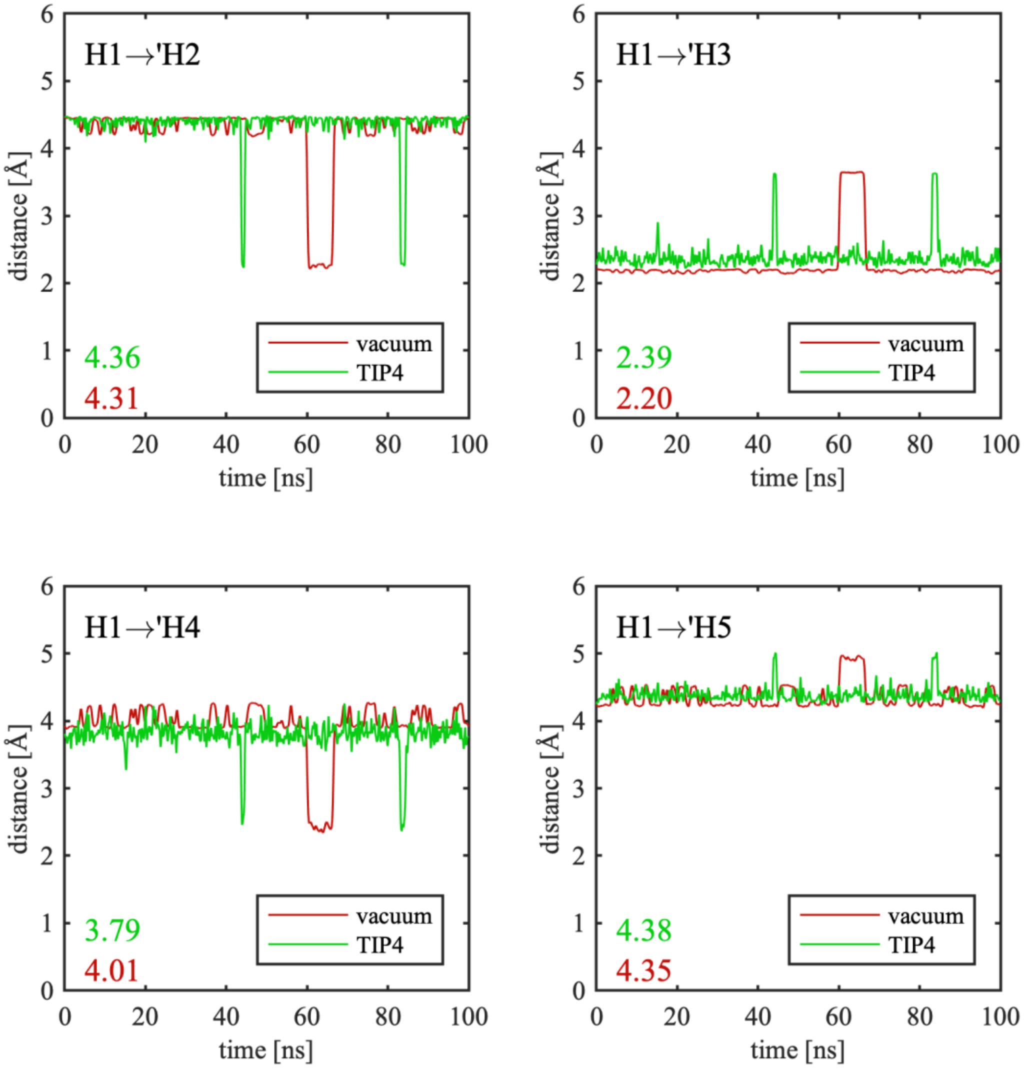

Figure S8. Interatomic distances for α-D-Gal*p*-(1→3)-α-D-Glc*p*.

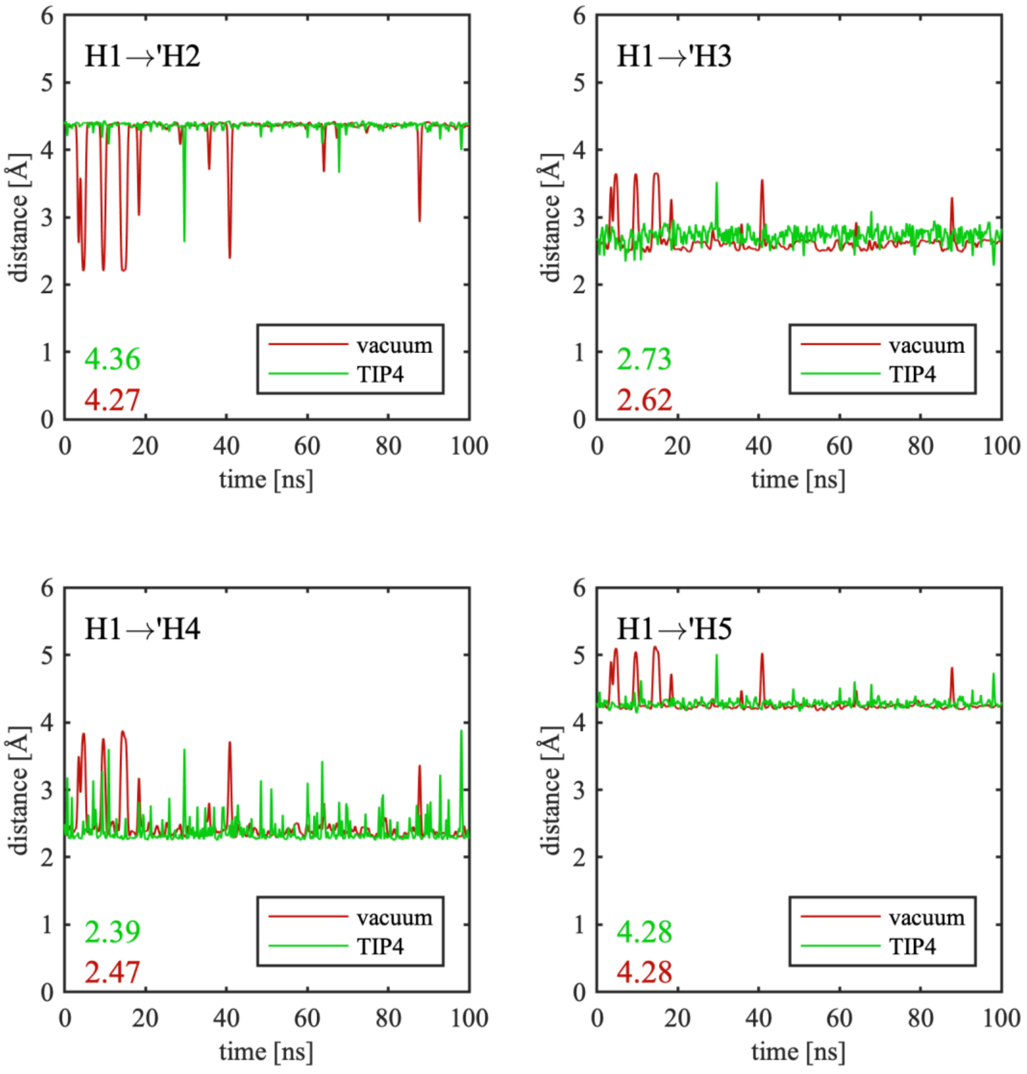

Figure S9. Interatomic distances for α-D-Glc*p*-(1→3)-α-D-Gal*p*.

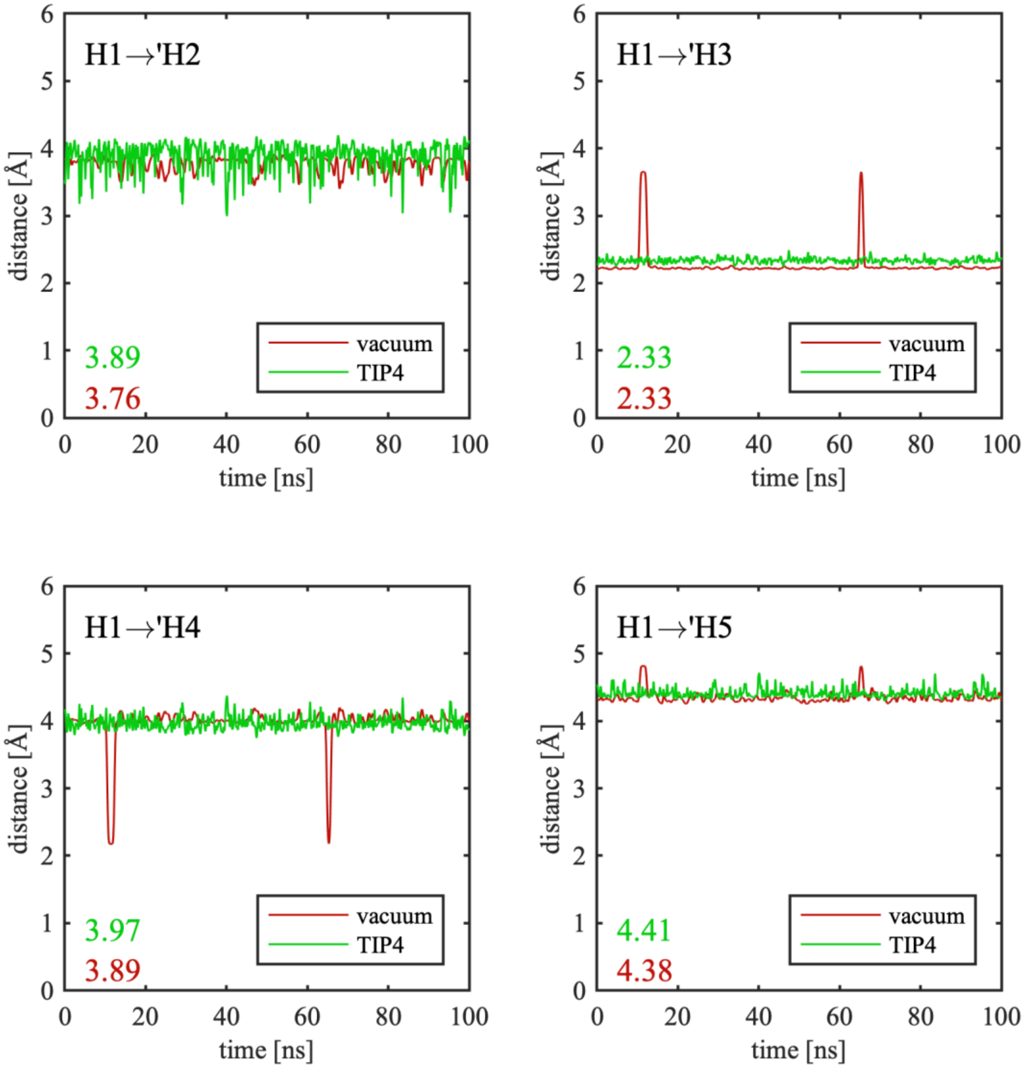

Figure S10. Interatomic distances for α-D-Gal*p*-(1→3)-α-D-Man*p*.

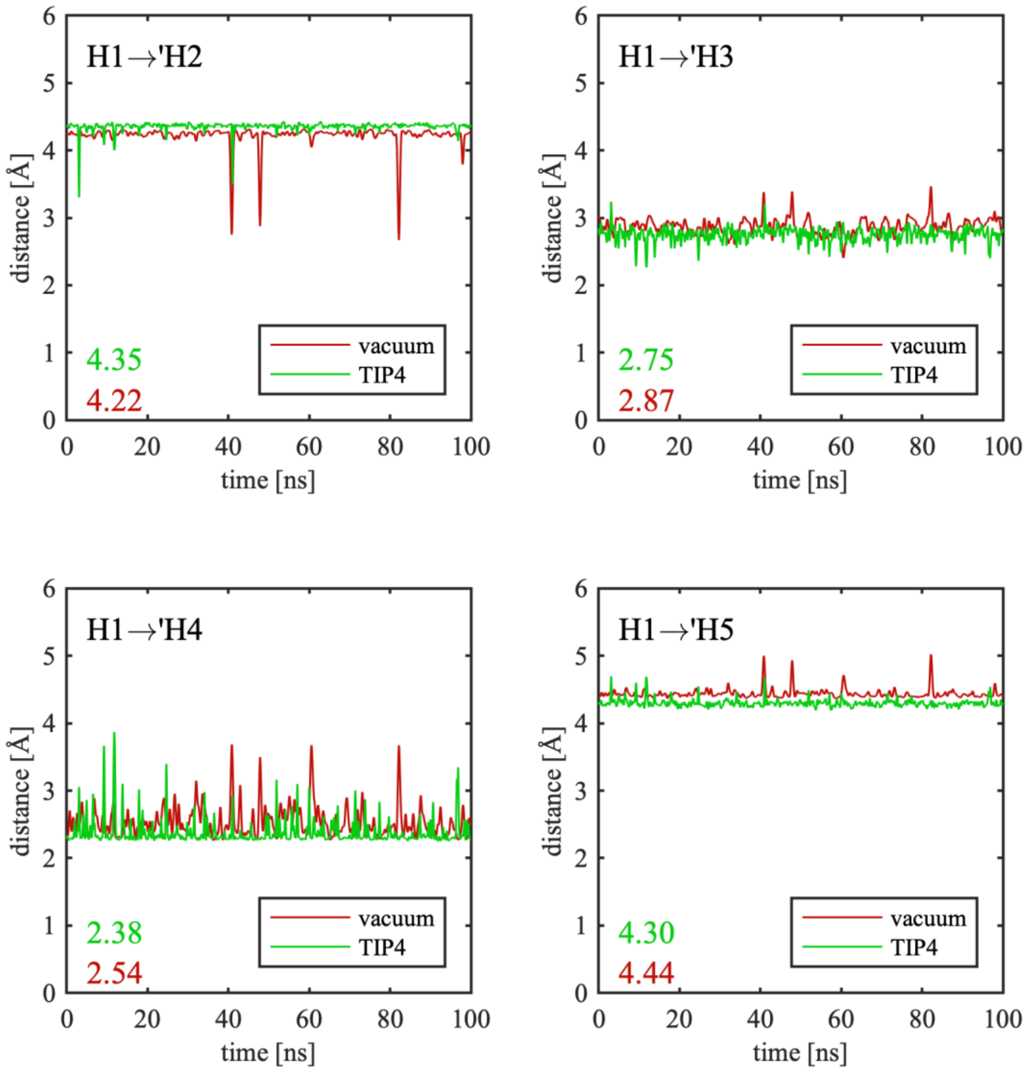

Figure S11. Interatomic distances for α-D-Man*p*-(1→3)-α-D-Gal*p*.
